# Oxytocin Intervention Mitigates Pathological and Behavioral Impairments in APP/PS1 Mice Subjected to Early Social Isolation

**DOI:** 10.1111/cns.70511

**Published:** 2025-07-10

**Authors:** Junjun Li, Yue Li, Zhuoya Wang, Yuan Yao, Dezhong Yao, Ke Chen, Yang Xia

**Affiliations:** ^1^ Department of Neurosurgery, Sichuan Provincial People's Hospital, MOE Key Lab for Neuroinformation, School of Life Science and Technology University of Electronic Science and Technology of China Chengdu China; ^2^ School of Electrical and Information Engineering Zhengzhou University Zhengzhou China; ^3^ The Clinical Hospital of Chengdu Brain Science Institute The Fourth People's Hospital of Chengdu Chengdu China

**Keywords:** Alzheimer's disease, gut microbiota, oxytocin, pathological damage, prefrontal cortex, social isolation

## Abstract

**Background:**

Neuropsychiatric symptoms, such as anxiety and depression, are prevalent during the prodromal phase of Alzheimer's disease (AD). Social isolation (SI) has been implicated as a potential exacerbating factor for emotional disturbances in AD pathogenesis. Despite the well‐established role of oxytocin (OXT) in regulating social behavior and mental health, its function and mechanisms in alleviating AD‐related psychiatric symptoms remain poorly understood.

**Materials and Methods:**

We utilized a 12‐week SI model to assess its effects on anxiety, depression‐like behaviors, and social cognition in early‐stage AD mice. Through immunofluorescence, enzyme‐linked immunosorbent assay, and 16S rDNA sequencing, we examined the changes in AD pathology and gut microbiota composition induced by SI, as well as the effects of OXT intervention.

**Results:**

Our findings revealed that SI markedly intensified anxiety‐like behaviors, depression‐like phenotypes, and social cognitive impairments in AD mice. Mechanistically, SI resulted in decreased OXT levels and upregulated OXT receptor expression while also exacerbating AD‐related pathological features, including increased Aβ plaque deposition, aberrant microglial proliferation, and reduced PSD‐95 expression in the prefrontal cortex. Furthermore, SI induced significant changes in gut microbiota composition. OXT intervention demonstrated therapeutic efficacy by mitigating behavioral deficits, alleviating AD‐related pathological damage, and restoring gut microbiota homeostasis in SI AD mice.

**Conclusion:**

These results underscore OXT as a promising therapeutic avenue for AD, offering novel insights into treatment strategies and identifying potential therapeutic targets through the restoration of gut homeostasis and mitigation of pathological processes.

## Graphical abstract

Social isolation exacerbates anxiety, depression, and social cognitive deficits in early‐stage AD mice by disrupting the oxytocin system, while intranasal oxytocin mitigates behavioral abnormalities, reduces pathological damage, and restores gut microbiota homeostasis.

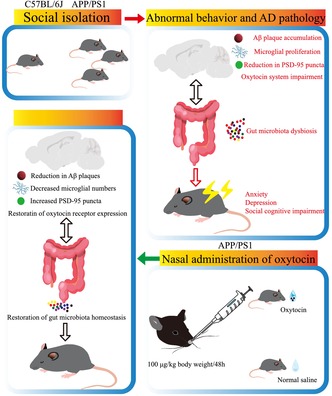

## 1 Introduction

Alzheimer's disease (AD) represents a devastating neurodegenerative disorder that poses a significant threat to the aging population, characterized by progressive neurological symptoms including anxiety, depression, memory loss, and cognitive decline [1]. In humans, social isolation (SI) is associated with a higher risk of mental health issues and increased mortality [2]. Emotional disturbance caused by loneliness and social disengagement is a significant risk factor for AD. These neuropsychiatric symptoms are considered both psychological responses to an AD diagnosis and early indicators of cognitive impairment [3, 4]. Despite extensive pharmacological research, AD remains largely intractable in clinical practice [5, 6]. Consequently, focusing on risk assessment and early intervention has emerged as a critical strategy for managing AD.

Oxytocin (OXT), a hormone and neurotransmitter composed of nine amino acids, is produced in the hypothalamus and stored in the posterior pituitary [7]. OXT serves dual roles as both a neurotransmitter and neuromodulator within the central nervous system (CNS) [8]. OXT plays a critical role in immune surveillance and maintaining immunological homeostasis when the organism faces diverse immune challenges, stressors, and tissue damage while also facilitating immune repair processes [9]. In stress‐induced inflammatory conditions, OXT mitigates the expression of pro‐inflammatory cytokines by modulating the hypothalamic–pituitary–adrenal axis and neuroinflammatory signaling pathways, such as those involving the CRHR1 protein [10]. Many of the protective effects of OXT observed throughout the lifespan are mediated by the OXT receptor (OXTR) [11]. The promoter region of the OXTR contains multiple binding sites for various transcription factors, which can regulate OXTR expression [12]. In a mouse model of intracerebral hemorrhage, OXTR expression in the striatum is upregulated, suggesting that OXTR signaling may serve as a compensatory response to injury through stimulation by its ligand, OXT [13, 14]. We hypothesize that OXT may alleviate emotional and physiological dysfunctions induced by social isolation stress via its immunomodulatory actions.

Evidence from patients and animal models indicates that prefrontal cortex (PFC) dysfunction contributes to the progression of AD [15, 16, 17]. Dysregulation of synaptic transmission in the PFC is strongly associated with social deficits, affective disorders, and memory loss in ad [ 18]. The PFC exhibits remarkable sensitivity to both stress and inflammation, which are primary etiological factors or exacerbating elements in the pathogenesis of most psychiatric disorders. In adult rats, 35 days of SI resulted in approximately a 15% increase in PFC Iba‐1‐positive protein expression [19]. A study revealed that anxiety levels are negatively correlated with the gray matter volume of the PFC and the alpha diversity index of the gut microbiome [20]. The gut microbiota interacts with the host brain and plays a pivotal role in the pathogenesis of neuropsychiatric disorders [21]. OXT can enhance gut barrier function, regulate intestinal inflammatory responses, and improve host health [22]. Compared to healthy controls, alterations in the composition and function of the gut microbiome have been observed in both AD patients and animal models [23]. Although existing studies have explored the effects of OXT on gastrointestinal function, its direct impact on gut microbiota composition in AD model animals, particularly under conditions of SI, remains insufficiently investigated.

In this study, we hypothesized that SI‐induced behavioral deficits are mediated through the oxytocinergic system dysregulation, leading to concurrent perturbations in both CNS homeostasis and gut microbiota composition in AD mouse models. To test this hypothesis, we employed intranasal OXT administration as an experimental intervention to evaluate the therapeutic potential for reversing SI‐induced AD‐related behavioral pathologies and to delineate the underlying neurobiological mechanisms.

## 2 Materials and Methods

### 2.1 Animals and Experimental Paradigms

Male C57BL/6J mice (*n* = 18) and APP/PS1 transgenic mice (*n* = 42), aged 4 weeks, were procured from Cavens Laboratory Animal Co. Ltd. (Changzhou, China). Mice were randomly assigned to either social isolation (SI; 1 mouse per cage; *n* = 9 per group; C57‐SI or APP/PS1‐SI) or group housing (GH; 3 mice per cage; *n* = 9 per group; C57‐GH or APP/PS1‐GH). For the OXT intervention experiment, socially isolated APP/PS1 mice were divided into two groups: one receiving intranasal OXT (1 mouse per cage; *n* = 12; OXT+APP/PS1‐SI) and the other receiving intranasal saline (NaCl) as a vehicle control (1 mouse per cage; *n* = 12; NaCl+APP/PS1‐SI). All animals were housed under controlled conditions (temperature: 22° ± 1°C; humidity: 55% ± 5%; 12‐h light–dark cycle) with ad libitum access to food and water.

**FIGURE 1:**
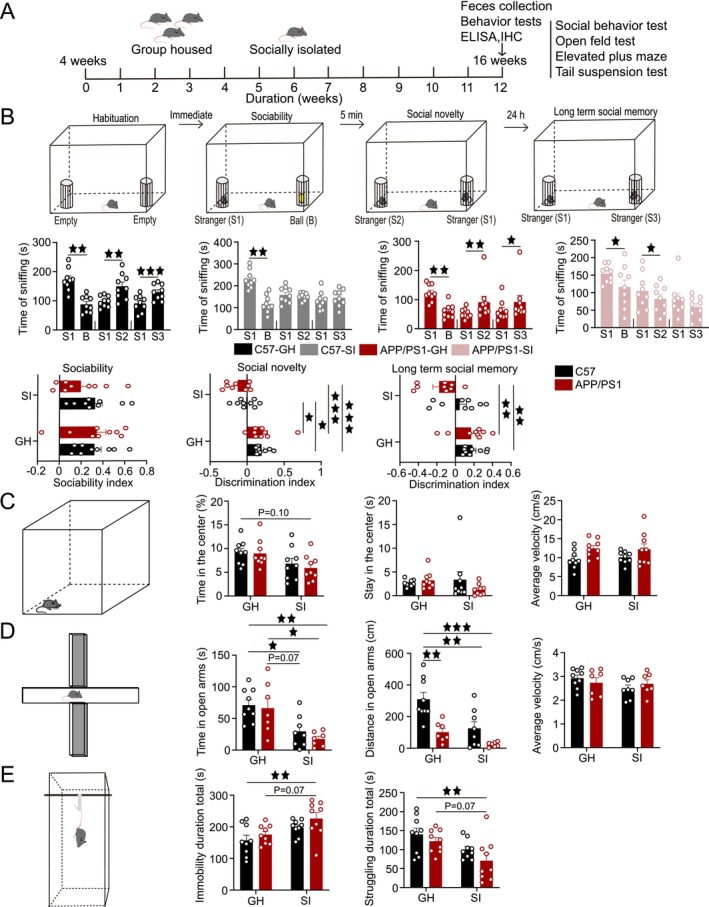
SI exacerbates behavioral deficits. (A) Experimental design schematic. (B) Schematic of the SBT. Sniffing time in sociability, social novelty, and long‐term social memory tests (paired *t*‐test). Sociability index and discrimination indices for social novelty and long‐term social memory. (C) Schematic of the OFT. Time in the center area, stay time in the central area, and movement speed in the OFT. (D) Schematic of the EPM. Time in open arms, distance traveled, and movement speed in the EPM. (E) Schematic of the TST. Immobility time and struggling time in the TST. Data was analyzed by two‐way ANOVA with Tukey's post hoc test; *n* = 7–9 per group. Data expressed as mean ± SEM. ^★^ *p* < 0.05, ^★★^ *p* < 0.01, ^★★★^ *p* < 0.001.

The experimental protocol began at 4 weeks of age, with SI and GH mice maintained under their respective housing conditions until 16 weeks. During the intervention phase, socially isolated APP/PS1 mice received eight intranasal administrations of OXT or NaCl, concluding at 16 weeks. At the endpoint, fresh fecal samples were collected for gut microbiota analysis. After behavioral tests, mice were anesthetized with pentobarbital sodium, euthanized, and brain tissues were harvested for enzyme‐linked immunosorbent assay (ELISA) and immunofluorescence (IF) staining (Figure 1A). All procedures followed guidelines approved by the Animal Ethics Committee of the University of Electronic Science and Technology of China.

### 2.2 Drug Administration

OXT, obtained from MedChemExpress Inc. (New Jersey, USA), was dissolved in 0.9% NaCl, aliquoted, and stored at −80°C until use. Mice received intranasal administration of either OXT (100 μg/kg) or an equivalent volume of saline (vehicle control). After 9 weeks of social isolation, the mice were subjected to 3 days of adaptive training. Intranasal interventions were then administered every 48 h, totaling eight administrations over the study duration.

### 2.3 Behavior Test

Before behavioral testing, animals were acclimated to the sound‐attenuated testing environment for at least 1 h to reduce stress and ensure consistent baseline conditions. Illumination was maintained at approximately 100 lx throughout all tests to ensure experimental uniformity [24]. To eliminate olfactory cues, all testing apparatuses were cleaned with 75% ethanol between trials and allowed to dry completely before reuse.

#### 2.3.1 Social Behavior Test

The social behavior test (SBT) adopts the established experimental paradigm [25]. The experiment comprised four phases: habituation, sociability, social novelty, and long‐term social memory. Test mice were introduced into the behavioral chamber (40 × 60 × 40 cm) from the same starting position, facing the wall. During the habituation phase, an empty cup was placed on each side of the chamber, and the test mouse was allowed to explore freely for 10 min before being removed. In the sociability phase, a juvenile mouse (stimulus, 3–4 weeks old) was placed under one cup, while a small ball was placed under the other. The test mouse was reintroduced to explore for 10 min and then removed. After a 5‐min interval, the social novelty phase began, with the familiar juvenile mouse placed under a cup on a different side and a novel juvenile mouse under the opposite cup. The test mouse was returned to the chamber for 10 min to assess its ability to distinguish between the familiar and novel mice. The long‐term social memory phase was conducted 24 h later, with the familiar juvenile mouse repositioned and a new novel mouse introduced. The test mouse's ability to differentiate was evaluated over 10 min. To reduce novelty‐induced stress, all juvenile stimulus mice were habituated under the cups for 30 min 1 day before testing. Sniffing duration and movement distance were recorded and analyzed using SMART v3.0 software (Panlab S.L.U., Spain). The discrimination index was calculated using the raw sniffing time data according to the following formula:

$$\begin{array}{c} \;\text{Sociability Index}\;\left(\mathrm{SI}\right)\\ =\frac{\text{time exploring social}\;\left(\mathrm{tS}\right)-\text{time exploring non social}\;\left(\mathrm{tNS}\right)\;}{\text{total exploration time}\;\left(\mathrm{tS}+\mathrm{tNS}\right)} \end{array}$$

$$\begin{array}{c} \text{Discrimination Index}\;\left(\mathrm{DI}\right)\\ =\frac{\text{time esploring novel}\;\left(\mathrm{tN}\right)-\text{time exploring familiar}\;\left(\mathrm{tF}\right)\;}{\text{total exploration time}\;\left(\mathrm{tN}+\mathrm{tF}\right)} \end{array}$$

#### 2.3.2 Open Field Test

The Open Field Test (OFT) was conducted in a 50 × 50 × 50 cm arena. Mice were placed facing the wall to minimize bias, and behavior was recorded for 5 min using an overhead camera. Data were analyzed using SMART v3.0, quantifying time in the central area (25 × 25 cm), stay time in the central area, and average velocity.

#### 2.3.3 Elevated Plus Maze

In the elevated plus maze (EPM), the mouse was gently placed at the central junction of the maze, facing an open arm, and its exploratory behavior was recorded for 5 min using an overhead camera. SMART v3.0 software automatically analyzed the time spent and distance traveled in the open arms, and average velocity.

#### 2.3.4 Tail Suspension Test

In the tail suspension test (TST), a 1 cm‐wide adhesive tape was attached 1 cm from the tip of the mouse's tail, and the mouse was suspended from a fixed stand. Behavioral data during the 5‐min test were recorded and analyzed using SMART v3.0 software to quantify the durations of immobility and struggling, thereby evaluating anxiety‐ and depression‐like behaviors.

### 2.4 Immunofluorescence Staining

Brain tissue samples were fixed in 4% PFA for 24 h, dehydrated in 15% and 30% sucrose solutions, and sectioned coronally at 30 μm using a cryostat. Sections were rinsed three times with 0.1 M PBS, blocked for 1 h in 0.3% Triton X‐100 and 3% BSA, and incubated with primary antibodies overnight at 4°C. Afterward, sections were treated with secondary antibodies and incubated at room temperature in the dark for 1 h. Nuclei were counterstained with DAPI for cellular localization. Fluorescence imaging was performed using a BX53F2 microscope (Olympus, Japan), and quantitative analyses were conducted using ImageJ. Antibody details are provided in Table S1.

### 2.5 Enzyme‐Linked Immunosorbent Assay

OXT concentrations in serum, intestinal tissue, and the PFC were measured using a commercial mouse OXT ELISA kit (ml002099, Shanghai Enzyme‐linked Biotechnology Co. Ltd.). Intestinal and brain tissues were homogenized in PBS at a 1:9 (w/v) tissue‐to‐PBS ratio. Blood samples were collected from the retro‐orbital sinus, clotted at room temperature, and centrifuged to isolate serum. Absorbance at 450 nm was measured using a Rayto RT‐6100 microplate reader (Rayto Life and Analytical Sciences Co. Ltd., China) following the manufacturer's protocol. Serum OXT levels were reported in pg/mL, while tissue OXT concentrations were normalized to total protein content and expressed as pg/mg protein.

### 2.6 16S rDNA Gene Sequencing and Analysis

After the completion of SI or GH feeding, fresh fecal samples were collected from the mice. A total of 6–8 fecal pellets were collected and placed in a 1.5 mL tube, then stored at −80°C until DNA extraction. Genomic DNA was extracted from the samples, and the V3‐V4 hypervariable regions of the 16S rDNA gene were amplified using barcode‐specific primers. Sequencing was performed on the Illumina MiSeq platform. The primers used for amplification were:

- 341F: CCTACGGGNGGCWGCAG.
- 806R: GGACTACHVGGGTATCTAAT.

The sequencing and subsequent analysis were carried out by GENEDENOVO Biotechnology (Guangzhou, China).

### 2.7 Statistical Analysis

Data are presented as mean ± SEM and analyzed using GraphPad Prism 9 (GraphPad Software, CA, USA). Normality was assessed prior to statistical analysis. For normally distributed data, two‐group comparisons were performed using a parametric t‐test, while non‐normally distributed data were analyzed with the Wilcoxon‐Mann–Whitney test. Differences among four groups were evaluated using two‐way ANOVA with Tukey's post hoc test. A P‐value < 0.05 was considered statistically significant. Each indicator was normalized individually, and group means were calculated. The Euclidean distance to the C57‐GH group was determined using the following formula:

$$d=\sqrt{\sum_{i=1}^{n}\;{\left(x_{i}-y_{i}\right)}^{2}}$$

where, *d*, Euclidean distance; *x*_i_, Normalized mean value of the i‐th indicator for the group being compared; *y*_i_, Normalized mean value of the i‐th indicator for the C57‐GH group; *n*, Total number of indicators.

All measurements and analyses were conducted in a blinded manner to ensure objectivity.

## 3 Results

### 3.1 SI Exacerbates Behavioral Deficits and AD‐Related Pathological Damage

In the sociability test, all groups—C57‐GH, C57‐SI, APP/PS1‐GH, and APP/PS1‐SI—exhibited a significant preference for conspecifics (*p* < 0.01, *p* < 0.01, *p* < 0.01, *p* < 0.05, respectively). During the social novelty test, the C57‐GH and APP/PS1‐GH groups showed a preference for the novel mouse (*p* < 0.01, *p* < 0.01), while the C57‐SI group lost this preference and the APP/PS1‐SI group favored the familiar mouse (*p* < 0.05). In the long‐term social memory test, the C57‐GH and APP/PS1‐GH groups sniffed the novel mouse more frequently (*p* < 0.001, *p* < 0.05), whereas the C57‐SI and APP/PS1‐SI groups displayed no preference. No significant differences were observed in the sociability index among groups. However, the social novelty discrimination index was higher in the C57‐GH group compared to the C57‐SI and APP/PS1‐SI groups (*p* < 0.05, *p* < 0.001), and the APP/PS1‐GH group also scored higher than the C57‐SI and APP/PS1‐SI groups (*p* < 0.05, *p* < 0.001). In the long‐term social memory test, the discrimination indices of the C57‐GH and APP/PS1‐GH groups were significantly higher than those of the APP/PS1‐SI group (*p* < 0.01, *p* < 0.01; Figure 1B).

In emotional behavioral assessments, the APP/PS1‐SI group showed a trend toward reduced time in the center area of the OFT compared to the C57‐GH group (*p* = 0.10). There was no significant difference in the residence time and movement speed of the four groups (Figure 1C). In the EPM, both the C57‐SI and APP/PS1‐SI groups spent significantly less time in the open arms than the C57‐GH group (*p* < 0.05, *p* < 0.01) and the APP/PS1‐GH group (*p* = 0.07, *p* < 0.05). The distance traveled in the open arms was also significantly reduced in the APP/PS1‐GH, C57‐SI, and APP/PS1‐SI groups compared to the C57‐GH group (*p* < 0.01, *p* < 0.01, *p* < 0.001), with no differences in movement speed (Figure 1D). In the TST, the APP/PS1‐SI group exhibited significantly longer immobility time (*p* < 0.01, *p* = 0.07) and shorter struggling time (*p* < 0.01, *p* = 0.07) compared to the C57‐GH and APP/PS1‐GH groups (Figure 1E).

**FIGURE 2:**
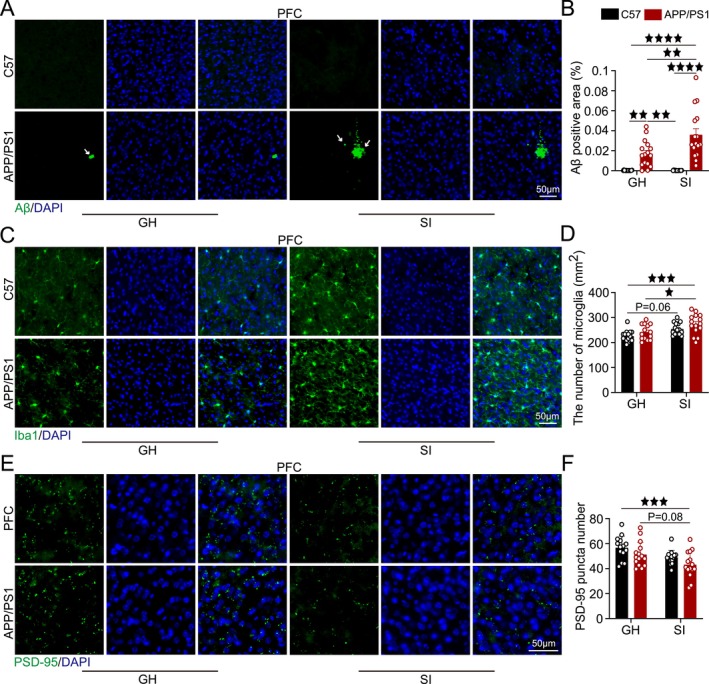
SI accelerates AD‐related pathological damage. (A) Representative images of Aβ (green) and DAPI (blue) co‐localization in the PFC, with statistical analysis of Aβ plaque area. (B) Representative images of Iba1 (green) and DAPI (blue) co‐localization in the PFC, with statistical analysis of Iba1‐positive cell counts. (C) Representative images of PSD‐95 (green) and DAPI (blue) co‐localization in the PFC, with quantitative analysis of PSD‐95 particle counts. Scale bar = 50 μm. *n* = 6, with 2–3 brain sections per mouse. Data analyzed by two‐way ANOVA with Tukey's post hoc test. Data expressed as mean ± SEM. ^★^ *p* < 0.05, ^★★^ *p* < 0.01, ^★★★^ *p* < 0.001, ^★★★★^ *p* < 0.0001.

Staining results showed that by 4 months of age, the APP/PS1‐GH group already displayed Aβ plaque deposition in the PFC, with significantly larger plaque areas compared to the C57‐GH and C57‐SI groups (*p* < 0.01, *p* < 0.01). The APP/PS1‐SI group exhibited a substantial increase in Aβ plaque area relative to the C57‐GH, APP/PS1‐GH, and C57‐SI groups (*p* < 0.0001, *p* < 0.01, *p* < 0.0001; Figure 2A). Microglial cell counts were significantly higher in the APP/PS1‐SI group compared to the C57‐GH and APP/PS1‐GH groups (*p* < 0.001, *p* < 0.05), with the C57‐SI group showing an upward trend relative to the C57‐GH group (*p* = 0.06; Figure 2B). Additionally, PSD‐95 expression in the APP/PS1‐SI group was significantly reduced compared to the C57‐GH group (*p* < 0.001) and showed a declining trend relative to the APP/PS1‐GH group (*p* = 0.08; Figure 2C).

### 3.2 SI Alters the Composition of Gut Microbiota in AD

**FIGURE 3:**
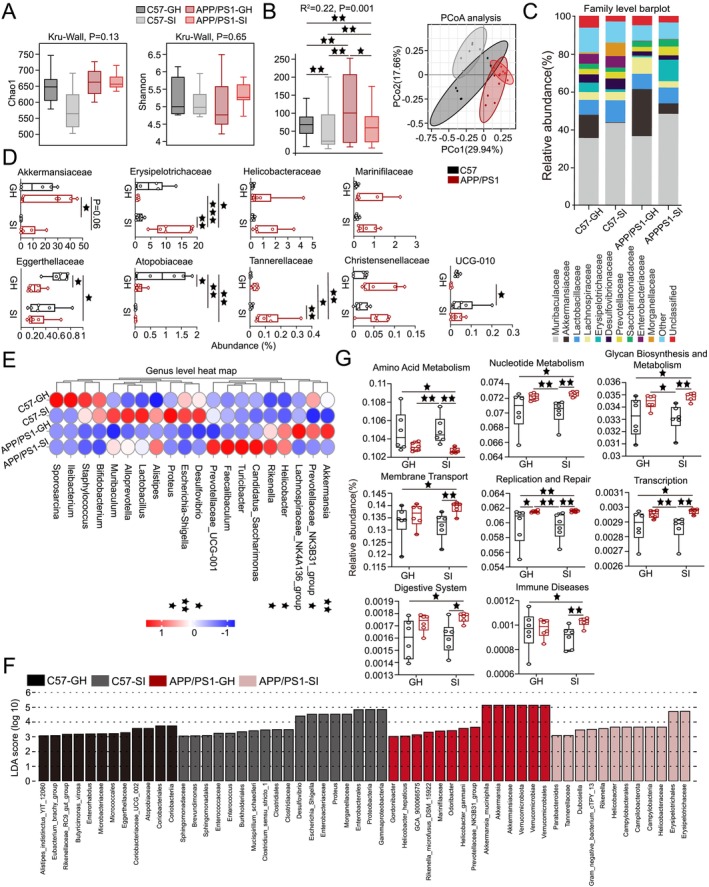
SI alters the composition of gut microbiota in AD. (A) α‐Diversity was assessed using the Chao1 richness estimator and Shannon diversity index. (B) PCoA of gut microbiota at the OTU level, with Adonis (PERMANOVA) test for *R* ^2^ and *p*‐values. (C) Species composition at the family level across groups. (D) Significant differences in gut microbiota at the family level. (E) Species composition at the genus level and inter‐group differences in gut microbiota. (F) Bar plot of LDA values showing differentially abundant microbiota (LDA score > 3). (G) Functional differences between groups based on Tax4Fun statistical testing. Data expressed as mean ± SEM; *n* = 6 per group. Kruskal–Wallis rank sum test was used for statistical analysis. ^★^ *p* < 0.05, ^★★^ *p* < 0.01, ^★★★^ *p* < 0.001.

Gut dysbiosis has been linked to the exacerbation of AD pathology. To investigate the impact of lifestyle and disease factors on gut microbiota composition and identify potential microbial biomarkers, we analyzed gut microbial profiles across experimental groups. α‐Diversity analysis, assessed using the Chao1 richness index and Shannon diversity index, showed no significant differences among the four groups (Figure 3A). In contrast, β‐diversity analysis, conducted through principal coordinate analysis (PCoA) combined with Adonis (PERMANOVA) testing, revealed significant differences in gut microbial community structure between groups (*p* < 0.01; Figure 3B).

At the family level, significant differences in gut microbial abundance were observed across experimental groups. Akkermansiaceae abundance was reduced in the APP/PS1‐SI and C57‐SI groups compared to the APP/PS1‐GH group (*p* = 0.06, *p* < 0.05). In contrast, Erysipelotrichaceae abundance was significantly higher in the APP/PS1‐SI group than in the C57‐GH, APP/PS1‐GH, and C57‐SI groups (*p* < 0.05, *p* < 0.001, *p* < 0.01). Eggerthellaceae abundance was significantly lower in the APP/PS1‐SI and APP/PS1‐GH groups compared to the C57‐GH group (*p* < 0.05, *p* < 0.05). The C57‐GH group exhibited higher Atopobiaceae abundance than the APP/PS1‐GH, C57‐SI, and APP/PS1‐SI groups (*p* < 0.05, *p* < 0.01, *p* < 0.01). Tannerellaceae abundance in the APP/PS1‐SI group was significantly reduced compared to the C57‐GH, APP/PS1‐GH, and C57‐SI groups (*p* < 0.01, *p* < 0.05, *p* < 0.01). Additionally, UCG‐010 abundance in the C57‐SI group was significantly higher than in the APP/PS1‐GH group (*p* < 0.05; Figure 3C,D). Notably, a significant correlation was identified between behavioral outcomes and gut microbiota abundance (Figure S1). At the genus level, among taxa with an abundance exceeding 0.1%, significant inter‐group differences were observed in the abundance of Akkermansia, Prevotellaceae_NK3B31_group, Helicobacter, Rikenella, Desulfovibrio, Escherichia‐Shigella, and Proteus (Figure 3E). The detailed results demonstrating these differences are shown in Figure S2.

LEfSe analysis (Linear Discriminant Analysis Effect Size) was used to identify differentially abundant microbiota with an LDA score > 3. Bar lengths represent the effect size of each microbial group, highlighting potential biomarkers (Figure 3F). Functional profiling via Tax4Fun statistical testing and STAMP analysis revealed significant differences in functional categories across groups at various KEGG classification levels. Key differences were observed in pathways related to amino acid metabolism, nucleotide metabolism, glycan biosynthesis and metabolism, membrane transport, replication and repair, transcription, digestive system, and immune diseases. Notably, the relative abundance of immune disease‐related pathways in the APP/PS1‐SI group was significantly higher compared to the C57‐GH and C57‐SI groups (*p* < 0.05, *p* < 0.01; Figure 3G).

### 3.3 SI Disrupts the OXT System in AD

**FIGURE 4:**
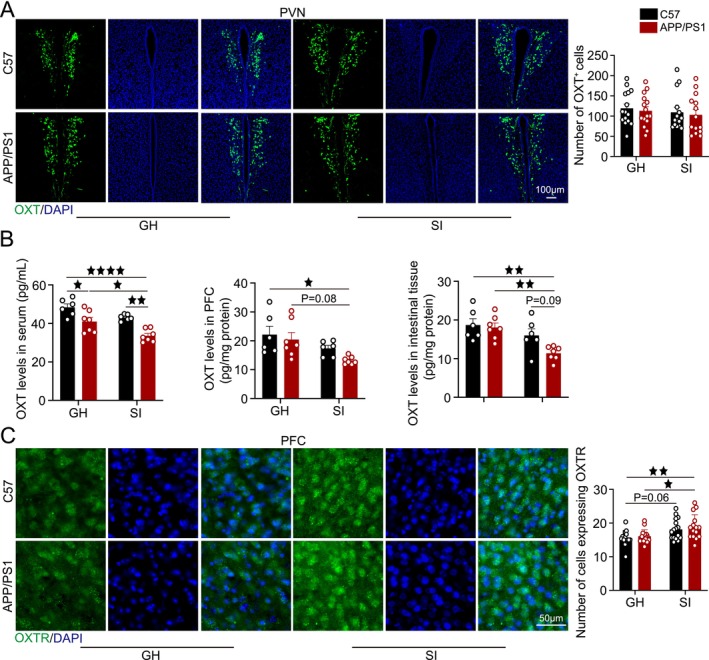
SI disrupts the OXT system. (A) Representative images of OXT neurons (green) co‐localized with DAPI (blue) in the PVN region, with quantitative OXT neuron counts. Scale bar = 100 μm. (B) OXT levels in serum, PFC, and intestinal tissue. (C) Representative images of OXTR‐positive neurons (green) co‐localized with DAPI (blue) in the PFC, with quantitative OXTR‐positive cell counts. Scale bar = 50 μm. Data was analyzed by two‐way ANOVA with Tukey's post hoc test; *n* = 6, with 2–3 brain sections per mouse. Data expressed as mean ± SEM. ^★^ *p* < 0.05, ^★★^ *p* < 0.01, ^★★★★^ *p* < 0.0001.

Then, we evaluated OXT neuron counts, OXT levels, and OXTR expression to investigate the impact of SI on the OXT system. No significant differences in OXT neuron counts were observed in the PVN across the four experimental groups (Figure 4A). OXT levels in serum, intestinal tissue, and the PFC were quantified. In serum, OXT levels in the APP/PS1‐SI group were significantly lower than those in the C57‐GH, APP/PS1‐GH, and C57‐SI groups (*p* < 0.0001, *p* < 0.05, *p* < 0.01), with the APP/PS1‐GH group also showing reduced levels compared to C57‐GH (*p* < 0.05). In the PFC, OXT levels in the APP/PS1‐SI group were significantly lower than in the C57‐GH and APP/PS1‐GH groups (*p* < 0.05, *p* = 0.08). In intestinal tissue, OXT levels in the APP/PS1‐SI group were markedly decreased compared to the C57‐GH, APP/PS1‐GH, and C57‐SI groups (*p* < 0.01, *p* < 0.01, *p* = 0.09; Figure 4B). OXTR expression in the PFC was significantly higher in the APP/PS1‐SI group compared to the C57‐GH and APP/PS1‐GH groups (*p* < 0.01, *p* < 0.05), with the C57‐SI group showing a trend toward increased expression relative to C57‐GH (*p* = 0.06; Figure 4C).

### 3.4 OXT Alleviates Emotional Disorders and Enhances Social Interaction

**FIGURE 5:**
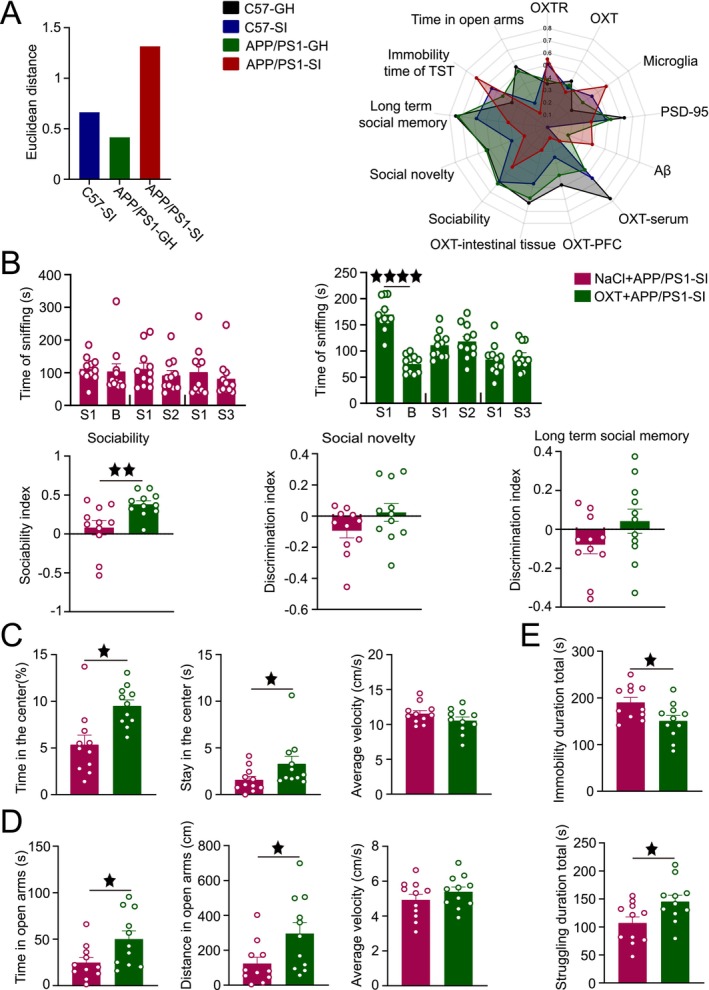
OXT alleviates emotional disorders and enhances social interaction. (A) Euclidean distances between the other three groups and the C57‐GH group and mean values of each indicator after normalization. (B) Sniffing time in the sociability, social novelty, and long‐term social memory tests (paired t‐test). Sociability index and discrimination indices for social novelty and long‐term social memory. (C) Time in the center area, stay time in the central area, and movement speed in the OFT (D) EPM results: Exploration time and travel distance in the open arms and average movement speed. (E) TST results: Struggling time and immobility time. *n* = 11. Unpaired *t*‐test. Data expressed as mean ± SEM. ^★^ *p* < 0.05, ^★★^ *p* < 0.01, ^★★★★^ *p* < 0.0001.

To quantify the effects of GH or SI lifestyle interventions, we employed Euclidean distance analysis. The results revealed that the APP/PS1‐SI group exhibited the greatest deviation from the C57‐GH group (*d* = 1.32), followed by the C57‐SI group (*d* = 0.66), while the APP/PS1‐GH group showed the smallest difference (*d* = 0.42). Radar charts were utilized to visually represent the differences among the four groups across multiple parameters (Figure 5A). Comprehensive behavioral, physiological, and pathological analyses revealed that SI exerted the most pronounced detrimental effects on AD mice.

Consequently, we implemented intranasal OXT administration in SI APP/PS1 mice and systematically evaluated its therapeutic efficacy. In the NaCl+APP/PS1‐SI group, mice showed no significant preference for sniffing stimulus mice during the sociability test and no significant differences in sniffing time between familiar and novel mice during the social novelty and long‐term social memory tests. In contrast, the OXT+APP/PS1‐SI group exhibited enhanced sociability, with a significantly stronger preference for sniffing stimulus mice (*p* < 0.0001). However, during the social novelty test, the preference for sniffing novel mice remained insignificant, and no significant differences in sniffing time were observed between familiar and novel mice in the long‐term social memory test. Analysis of the discrimination indices revealed that the sociability index in the OXT+APP/PS1‐SI group was significantly higher than that in the NaCl+APP/PS1‐SI group (*p* < 0.01). However, no significant differences were observed between the two groups in the social novelty and long‐term social memory discrimination indices (Figure 5B).

In the OFT, the OXT+APP/PS1‐SI group spent significantly more time in the center area and had longer center durations compared to the NaCl+APP/PS1‐SI group (*p* < 0.05, *p* < 0.05), with no significant difference in average movement speed (Figure 5C). In the EPM, the OXT+APP/PS1‐SI group showed significantly increased exploration time and travel distance in the open arms compared to the NaCl+APP/PS1‐SI group (*p* < 0.05, *p* < 0.05), with no significant difference in average movement speed (Figure 5D). In the TST, the OXT+APP/PS1‐SI group exhibited significantly higher struggling time and significantly lower immobility time compared to the NaCl+APP/PS1‐SI group (*p* < 0.05, *p* < 0.05; Figure 5E).

### 3.5 OXT Reduces OXTR Expression and AD‐Related Pathological Damage

**FIGURE 6:**
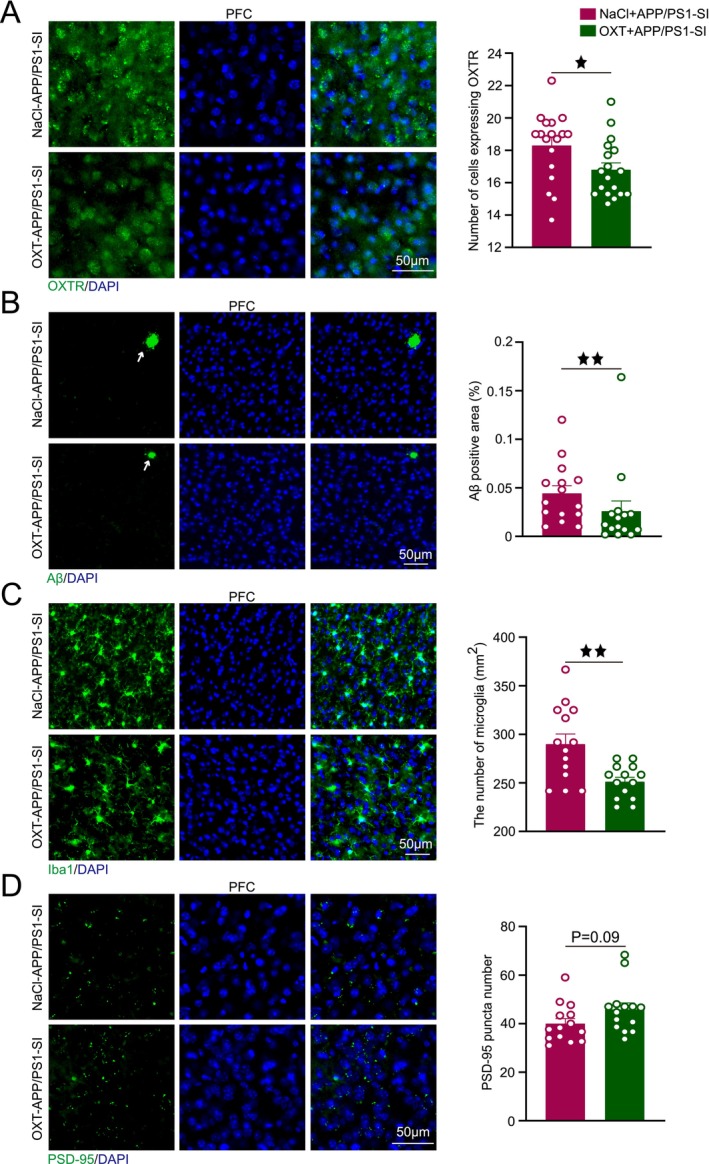
OXT reduces OXTR expression and AD‐related pathological damage. (A) Representative images of OXTR (green) and DAPI (blue) colocalization in the PFC post‐intervention, with quantitative analysis of OXTR‐positive cells. (B) Representative images of Aβ (green) and DAPI (blue) colocalization in the PFC post‐intervention, with quantitative analysis of Aβ plaque area. (C) Representative images of Iba1 (green) and DAPI (blue) colocalization in the PFC post‐intervention, with quantitative analysis of Iba1‐positive cells. (D) Representative images of PSD‐95 (green) and DAPI (blue) colocalization in the PFC post‐intervention, with quantitative analysis of PSD‐95 puncta. Scale bar = 50 μm. *n* = 6, with 2–3 brain sections per mouse. Data was analyzed using unpaired t‐tests and expressed as mean ± SEM. ^★^ *p* < 0.05, ^★★^ *p* < 0.01.

Following OXT intervention, we observed significant changes in key pathological markers in the PFC. The OXT+APP/PS1‐SI group exhibited significantly lower OXTR expression levels compared to the NaCl+APP/PS1‐SI group (*p* < 0.05; Figure 6A). Additionally, the Aβ plaque area in the OXT+APP/PS1‐SI group was substantially reduced, being significantly smaller than that in the NaCl+APP/PS1‐SI group (*p* < 0.01; Figure 6B). Similarly, microglial cell counts in the OXT+APP/PS1‐SI group were significantly lower than those in the NaCl+APP/PS1‐SI group (*p* < 0.01; Figure 6C). Furthermore, the number of PSD‐95 puncta in the PFC of the OXT+APP/PS1‐SI group showed an increasing trend compared to the NaCl+APP/PS1‐SI group (*p* = 0.09; Figure 6D).

### 3.6 OXT Regulates Gut Microbial Homeostasis

**FIGURE 7:**
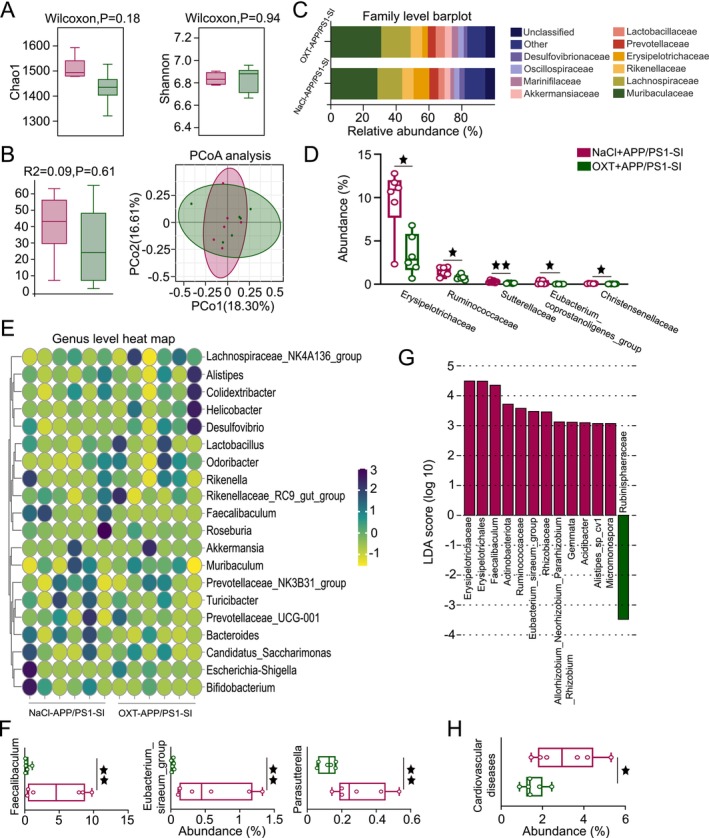
OXT regulates gut microbial homeostasis. (A) α‐Diversity of gut microbiota assessed using Chao1 richness and Shannon diversity indices. (B) PCoA at the OTU level, with Adonis (PERMANOVA) analysis for *R* ^2^ and *p*‐values. (C) Taxonomic composition of gut microbiota at the family level in both groups. (D) Differentially abundant gut microbiota at the family level. (E) Taxonomic composition of gut microbiota at the genus level in both groups. (F) Genus‐level gut microbiota showing significant intergroup differences. (G) Bar chart of LDA values. (H) Functional differences between groups were analyzed using PICRUSt2 statistical testing. *n* = 6. Wilcoxon rank‐sum test was used for statistical analysis. Data expressed as mean ± SEM. ^★^ *p* < 0.05, ^★★^ *p* < 0.01.

Fresh fecal samples were collected post‐intervention for 16S rDNA sequencing to investigate the regulatory effects of OXT on gut microbiota homeostasis. No significant differences in α‐diversity, assessed using the Chao1 richness index and Shannon diversity index, were observed between the NaCl+APP/PS1‐SI and OXT+APP/PS1‐SI groups (Figure 7A). Similarly, β‐diversity analysis via PCoA combined with Adonis (PERMANOVA) testing showed no significant differences between the two groups (Figure 7B).

At the family level, the relative abundances of Erysipelotrichaceae, Ruminococcaceae, Sutterellaceae, Eubacterium_coprostanoligenes_group, and Christensenellaceae were significantly lower in the OXT+APP/PS1‐SI group compared to the NaCl+APP/PS1‐SI group (Figure 7C,D). Correlations between behavioral changes and microbial abundance following OXT intervention were also identified (Figure S3). At the genus level, the OXT+APP/PS1‐SI group exhibited significantly reduced abundances of Faecalibaculum, Eubacterium_siraeum_group, and Parasutterella compared to the NaCl+APP/PS1‐SI group (Figure 7E,F).

Using LEfSe analysis, microbial taxa with an LDA score > 3 were visualized in bar plots, with bar length representing the effect size of each microbial group, identifying potential biomarkers (Figure 7G). Based on PICRUSt2 statistical analysis, OXT intervention significantly reduced the abundance of microbial taxa associated with cardiovascular diseases in the OXT+APP/PS1‐SI group compared to the NaCl+APP/PS1‐SI group (*p* < 0.05; Figure 7H). These findings suggest that OXT not only modulates gut microbiota composition but also reduces the risk of cardiovascular diseases in AD mice, underscoring its potential therapeutic benefits.

## 4 Discussion

Focusing on changes in gut microbiota and pathological alterations in the PFC, we investigated the mechanisms by which intranasal OXT administration alleviates neuropsychiatric disorders in AD mice following prolonged SI. Under SI conditions, mice exhibited both emotional disturbances and social cognitive impairments, with these effects being most pronounced in APP/PS1 mice. Intranasal OXT intervention effectively counteracted the accelerated AD progression induced by SI, a process associated with dynamic modulation of the OXT system, which may explain the observed behavioral and pathological changes. Our study elucidates the multifaceted impact of SI on AD‐vulnerable individuals, providing potential diagnostic insights. Importantly, our findings indicate that OXT may have both protective and restorative effects on central and peripheral systems in socially isolated AD model mice, providing a rationale for future translational research.

### 4.1 SI Accelerates the Progression of AD

The significance of social interaction is underscored by studies identifying SI and loneliness as risk factors for adverse health outcomes and increased mortality [26]. Both genetic mutations and environmental factors, such as chronic stress, are well documented to elevate the risk of developing ad [ 27]. In our study, SI induced aberrant neuropsychiatric symptoms in 4‐month‐old APP/PS1 mice, with C57 mice also exhibiting behavioral alterations. Among all group comparisons, Euclidean distance analysis revealed the greatest divergence between SI‐APP/PS1 mice and GH‐C57 controls. Previous research has shown that a lack of social interaction heightens the risk of mental disorders, triggering anxiety‐ and depression‐like behaviors [28, 29]. Mood disturbances, which primarily occur in the early stages of AD, represent emotional responses to progressive cognitive decline. Additionally, SI disrupts social cognition behaviors in mice. The study found that SI impairs social recognition, primarily due to damage to PFC‐related circuits [30]. We propose that SI extinguishes social curiosity in mice, and interactions with unfamiliar mice may induce social stress in SI mice, potentially explaining the observed impairment in social recognition.

SI accelerates Aβ accumulation in the PFC. Positron emission tomography imaging studies have demonstrated a significant correlation between Aβ burden and feelings of loneliness [31]. Intracerebroventricular injection of Aβ25‐35 oligomers has been shown to induce depression‐ and anxiety‐like behaviors in rats, indicating that brain Aβ levels significantly influence the psychiatric states of patients or model animals [32]. The production and accumulation of Aβ play a pivotal role in the interactions among oxidative stress, inflammation, and apoptosis [33]. In our experiment, abnormal microglial proliferation was observed in the PFC of SI APP/PS1 mice, which we hypothesize results from a cascade reaction triggered by the gradual accumulation and aggregation of Aβ peptides. Under depressive pathological conditions, activated microglia can aberrantly phagocytize neuronal synapses, leading to synaptic loss [34]. The gut microbiota is considered a critical component of host immune regulation [35]. Gut microbiota dysbiosis can lead to alterations in the intestinal epithelial barrier and promote inflammation by modulating cytokine activity [36]. Lifestyle and genotype significantly influence the β‐diversity of gut microbiota in mice, with profound effects on microbial composition at both the family and genus levels. Our findings demonstrate a significant correlation between gut microbiota composition and behavioral impairments, underscoring the pivotal role of the gut‐brain axis in mediating behavioral alterations. As in previous studies, emotional stress significantly impacts AD, with SI causing immune organ damage and abnormal behavioral manifestations in AD mice [37].

### 4.2 Role of OXT System in AD Pathogenesis

OXT plays a critical role in the pathogenesis of AD, particularly in regulating social interaction and emotional balance in neurodegenerative diseases. In our experiment, SI conditions did not significantly affect the number of OXT neurons in APP/PS1 mice, likely due to the distinct physiological responses of different neuron types to environmental and pathological factors. Studies on AD patients suggest that reduced activation, rather than loss, of OXT neurons may occur in AD brains [38]. In a chronic inflammatory environment, OXT secretion may be impaired [39]. We observed reduced OXT levels in serum, brain tissue, and intestinal tissue. Research indicates that lower OXT levels are closely associated with anxiety and depression and may impair social cognition and prosocial behavior [40]. Reduced OXT levels can exacerbate neuroinflammation and hinder the brain's ability to repair damage [41]. Additionally, decreased OXT levels may compromise the integrity of the gut immune barrier, leading to changes in gut microbiome composition and activity, ultimately causing dysbiosis [42]. Another component of the OXT system, OXTR, shows increased expression in response to elevated inflammation levels [43], consistent with our experimental findings. SI‐induced central and peripheral inflammation in AD mice may require increased OXTR activation to address immune challenges.

### 4.3 OXT as a Promising Therapeutic Intervention for AD

There is an urgent need for cost‐effective, low‐side‐effect interventions to prevent the onset and progression of ad [ 44]. OXT, a promising anti‐inflammatory therapeutic molecule, can modulate psychological and physical health changes induced by social environments, stress responses, and stress‐related factors [45]. We noted that, compared with the APP/PS1‐SI group, mice in the NaCl+APP/PS1‐SI group exhibited reduced time spent exploring conspecifics during the sociability phase. This may have resulted from mild stress responses triggered by repeated handling and intranasal administration, thereby affecting their sociability performance. However, no significant difference in the sociability index was observed between the two groups (Figure S4), suggesting that this effect does not compromise our conclusion regarding the ability of OXT to enhance sociability in AD mice.

Following intranasal OXT intervention, OXTR expression was significantly downregulated. This discovery suggests that OXT exerts regulatory effects on the OXT system, potentially mediating immunomodulatory effects on the organism. Furthermore, the suppression of abnormal microglial proliferation in the PFC brain region further supports this hypothesis. Previous studies have shown that intravenous injection of OXT, which specifically binds to pathologically upregulated OXTR, can reduce the levels of inflammatory cytokines and alleviate AD pathological damage by blocking the ERK/p38 MAPK and COX‐2/iNOS NF‐κB signaling pathways [46]. Experiments have confirmed that intranasal OXT administration reduces Aβ plaque area in the PFC. Intravenous tail vein administration of OXT in group‐housed 12‐week‐old APP/PS1 mice reduced age‐associated Aβ deposition and neuronal apoptosis, alleviated cognitive decline [46]. Intranasal administration of OXT after bilateral Aβ injection into the hippocampal CA1 region improved Aβ‐induced deficits in working and spatial memory and upregulated synaptic plasticity‐related and neurogenesis markers in male Wistar rats [47]. In aged APP/PS1 mice, OXT intervention inhibits microglial activation, reduces Aβ deposition in dense‐core plaques, and restores behavioral impairments [48]. This supports the notion that, independent of SI, OXT can exert beneficial effects on pathological and behavioral impairments across different stages of AD progression.

OXT can reduce the production of pro‐inflammatory factors, mitigate oxidative stress responses, and protect intestinal barrier integrity [22]. Ruminococcaceae, previously linked to depression [49, 50], showed reduced abundance in SI AD mice following OXT intervention. In a restraint stress model, the gut microbiota analysis showed an increased abundance of Sutterellaceae [51]. Similarly, in SI mice, OXT intervention also resulted in a reduction in the abundance of this microbiota. In summary, intranasal OXT intervention effectively improves neuropsychiatric symptoms in AD mice, alleviates AD‐related pathological burden, and restores gut microbiota homeostasis.

## 5 Conclusion

The regulation of emotional and social behaviors by OXT may represent an effective strategy for delaying AD progression. Future research should further explore the effects of different OXT administration methods and dosages to optimize intervention protocols. Neuropsychiatric disorders, as early warning indicators, play a critical role in the onset and progression of AD. Our findings not only reveal the detrimental effects of SI on AD but also provide novel insights into early intervention strategies. These results further expand the therapeutic potential of OXT, highlighting its significance in treating related disorders.

## Ethics Statement

The study was approved by the Ethics Committees of University of Electronic Science and Technology of China.

## Conflicts of Interest

The authors declare no conflicts of interest.

## Supporting information


Data S1.

## Data Availability

The data that support the findings of this study are available from the corresponding author upon reasonable request.
