# Supplementary material for: Oxytocin Intervention Mitigates Pathological and Behavioral Impairments in APP/PS1 Mice Subjected to Early Social Isolation
Source: CNS Neurosci Ther. 2025 Jul 10;31(7):e70511. doi: 10.1111/cns.70511 (PMC12241820; doi:10.1111/cns.70511)
Supplement: Supplementary file 1 — Data S1. [file CNS-31-e70511-s001.docx]

**Supporting Information**

**Table S1. Antibody Details in Immunofluorescence Staining Protocols**

| **Antibody type** | **Designation** | **Company** | **Cat#** | **Dilytion Rate** |
| --- | --- | --- | --- | --- |
| **Primary antibody** | anti-OXTR | Proteintech | 23045-1-AP | 1:100 |
|  | anti-Oxytocin-neurophysin 1 | Abcam | ab212193 | 1:400 |
|  | anti-β-Amyloid | Cell Signaling Technology | 8243S | 1:1000 |
|  | anti-PSD-95 | Cell Signaling Technology | 3450T | 1:250 |
|  | anti-Iba1 | Abcam | ab178847 | 1:100 |
| **Secondary antibody** | goat anti-rabbit IgGs | ThermoFisher | A-11008 | 1:500 |

**
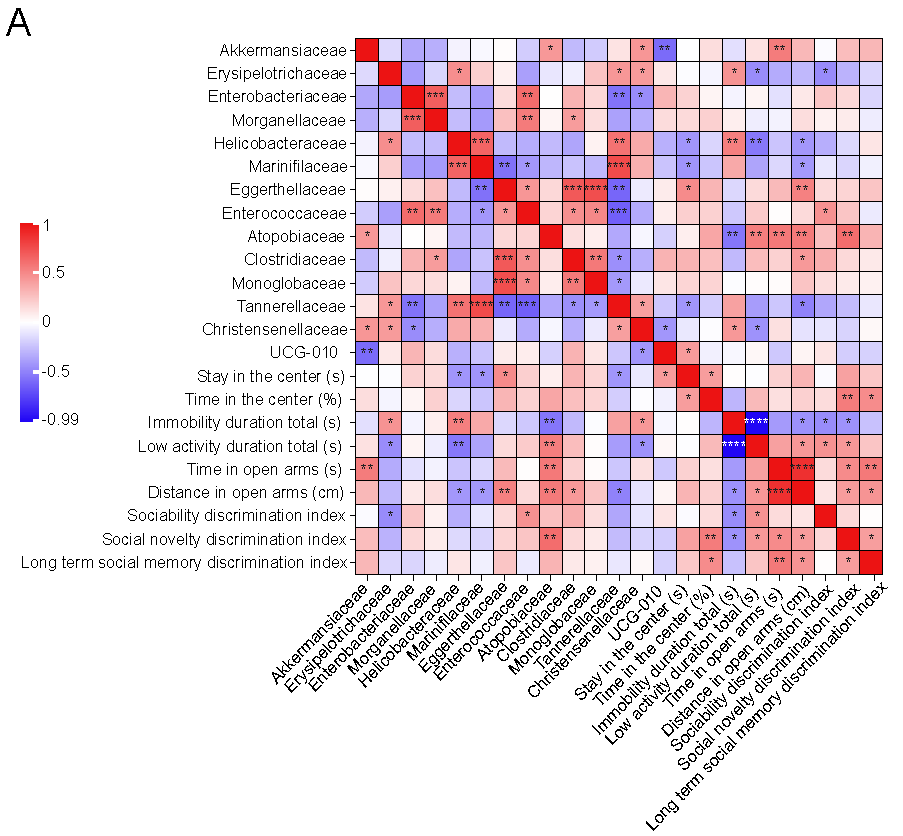
**

**Fig. S1. Analysis of the association between behavioral outcomes and intestinal microbial communities.** (A) Correlation analysis between the behavioral results of the C57-GH, C57-SI, APP/PS1-GH, and APP/PS1-SI groups and the gut microbiota abundance at the family level. Spearman correlation. ^★^P<0.05, ^★★^P<0.01, ^★★★^P<0.001, ^★★★★^P<0.0001.


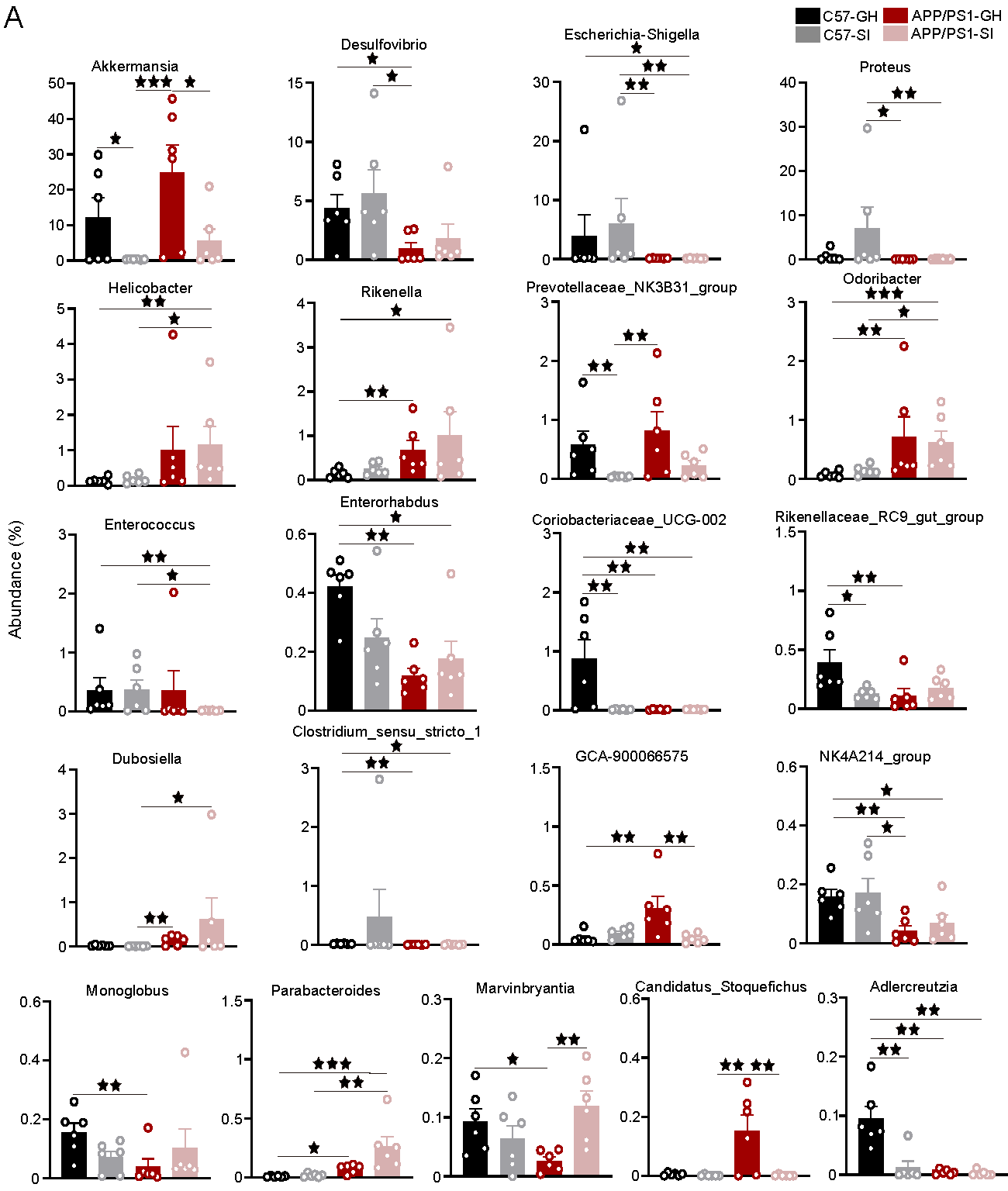


**Fig. S2. Statistical results of genus-level gut microbiota abundance following OXT intervention.** (A) Genus-level gut microbiota showing significant intergroup differences. n=6. Wilcoxon rank-sum test was used for statistical analysis. Data expressed as mean ± SEM. ^★^P<0.05, ^★★^P<0.01, ^★★★^P<0.001.


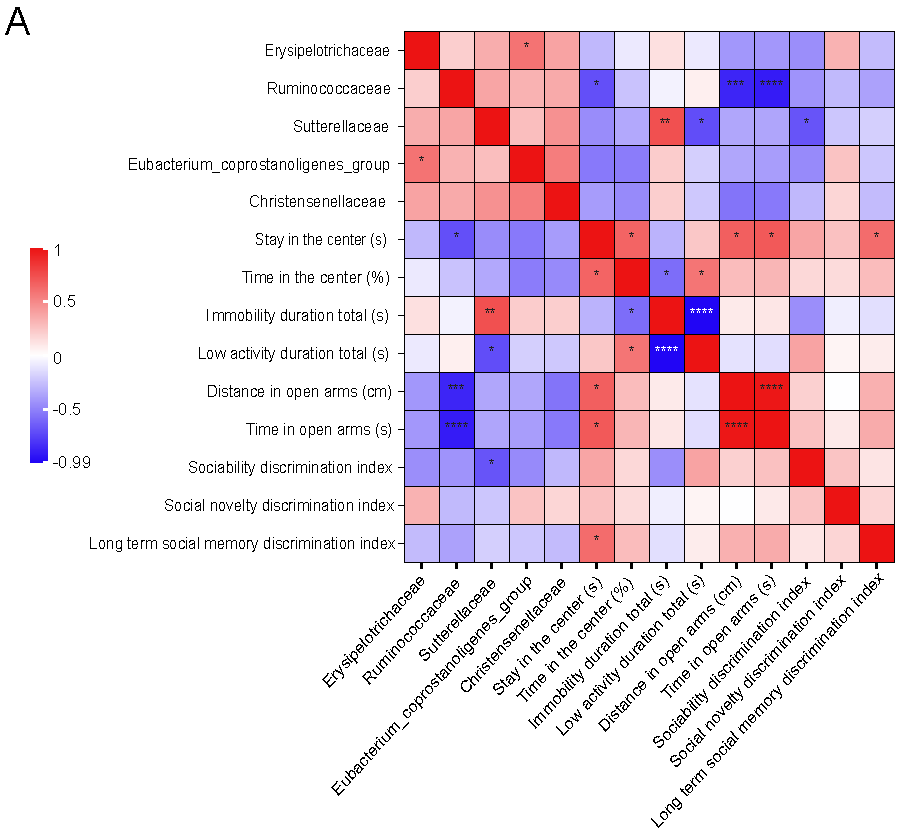


**Fig. S3. Analysis of the association between behavioral outcomes and intestinal microbial communities.** (A) Correlation analysis between the behavioral results of NaCl+APP/PS1-SI and OXT+APP/PS1-SI groups and the gut microbiota abundance at the family level. Spearman correlation. ^★^P<0.05, ^★★^P<0.01, ^★★★^P<0.001, ^★★★★^P<0.0001.


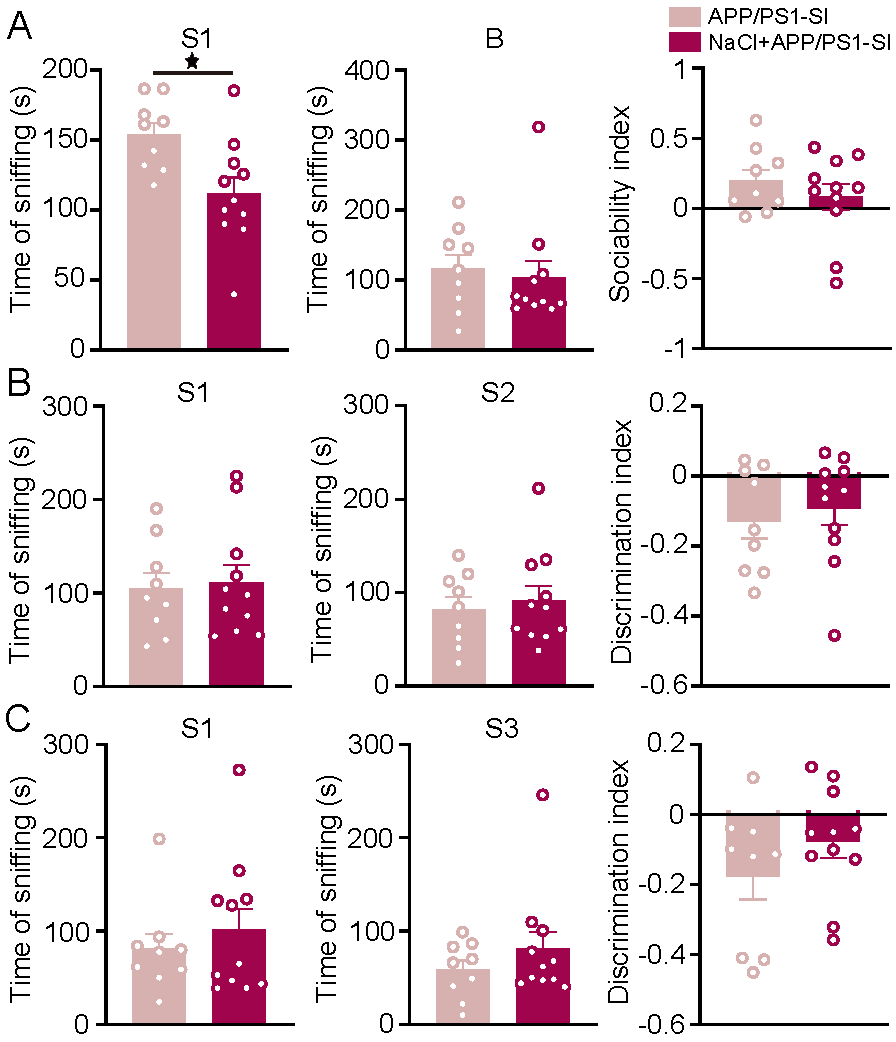


**Fig. S4. Comparison of social behavior at each stage between the APP/PS1-SI group and the NaCl+APP/PS1-SI group.** (A) Comparison of exploration time toward conspecific and ball, and sociability index across the sociability phase. (B) Comparison of exploration time toward familiar and novel conspecifics, and discrimination index in the social novelty phase. (C) Comparison of exploration time toward familiar and novel conspecifics, and discrimination index in the long-term social memory phase. n = 9 (APP/PS1-SI), 11 (NaCl+APP/PS1-SI). Unpaired t-test. Data expressed as mean ± SEM. ^★^P<0.05.
